# Hyperthyroidism Associated with Gestational Trophoblastic Neoplasia: Systematic Literature Review and Pathways Analysis

**DOI:** 10.3390/cancers17091398

**Published:** 2025-04-22

**Authors:** Alina Badlaeva, Anna Tregubova, Aleksandra Asaturova, Beatrice Melli, Vincenza Ylenia Cusenza, Andrea Palicelli

**Affiliations:** 1 1st Pathology Department, National Medical Research Center for Obstetrics, Gynecology and Perinatology Named After Academician V.I. Kulakov of the Ministry of Health of Russia, Bldg. 4, Oparina Street, Moscow 117513, Russia; alinamagnaeva03@gmail.com (A.B.); a_tregubova@oparina4.ru (A.T.); 2 Molecular Pathology, Azienda USL-IRCCS di Reggio Emilia, 42123 Reggio Emilia, Italy; beatrice.melli@ausl.re.it (B.M.); vincenzaylenia.cusenza@ausl.re.it (V.Y.C.); 3 Unit of Obstetrics and Gynecology, Azienda USL-IRCCS di Reggio Emilia, 42123 Reggio Emilia, Italy; 4 Pathology Unit, Azienda USL-IRCCS di Reggio Emilia, 42123 Reggio Emilia, Italy; andrea.palicelli@ausl.re.it; 5 Clinical and Experimental Medicine PhD Program, University of Modena and Reggio Emilia, 41121 Modena, Italy

**Keywords:** gestational trophoblastic disease, gestational trophoblastic neoplasia, molar pregnancy, hydatidiform moles, choriocarcinoma, placental site trophoblastic tumor, human chorionic gonadotropin, hyperthyroidism, thyroid storm

## Abstract

**Background/Objectives**: Gestational trophoblastic disease (GTD) is a group of disorders including complete, partial, and invasive/metastatic hydatidiform moles, as well as gestational trophoblastic neoplasia (GTN) (choriocarcinoma; placental site trophoblastic tumor, PSTT; epithelioid trophoblastic tumor, ETT; or mixed forms). These entities are characterized by increased trophoblast proliferation, rarely complicated by hyperthyroidism. **Methods**: Our systematic literature review (PRISMA guidelines; PubMed, Web of Science, and Scopus databases) searched for histologically confirmed cases of GTN associated with clinical or subclinical hyperthyroidism. We described the clinical–pathologic features and the pathways of hyperthyroidism in GTD. **Results**: We identified just 32 choriocarcinomas and one PSTT; other non-histologically confirmed cases could have been identified, as some patients received a clinical diagnosis based on serum human chorionic gonadotropin (hCG) levels and imagining data and were treated accordingly. As regards choriocarcinomas, patients’ age range was 15–45 (mean 27) years. Metastases involved the lungs (53%), brain (25%), and liver (19%) (less frequently, the kidneys, spleen, ovaries, vagina, pelvis/abdomen, or thyroid). The time to recurrence range was 1–36 (mean 12) months. On follow-up, 10 patients (32%) were alive with disease and 6 (19%) showed no evidence of disease, while most of the women (15 cases, 48%) died of disease. The hCG level range was 10,000–3,058,000,000 (mean 128,957,613) IU/L. At least some symptoms and/or signs of hyperthyroidism were evident with variable intensity in most cases and significantly improved within 2–3 weeks after treatment. **Conclusions**: Increased trophoblast proliferation could stimulate thyroid function via increasing the half-life of thyroxine-binding globulin. Secondly, increased hCG demonstrates cross-reactivity with the thyroid-stimulating hormone due to similar α-subunits. Moreover, basic isoforms of hCG may facilitate thyrotropic activity.

## Simple Summary

It is vitally important that scientists are able to describe their work simply and concisely to the public, especially in an open-access on-line journal. The simple summary consists of no more than 200 (80–150) words in one paragraph and contains a clear statement of the problem addressed, the aims and objectives, pertinent results, conclusions from the study and how they will be valuable to society. This should be written for a lay audience, i.e., no technical terms without explanations. No references are cited and no abbreviations. Submissions without a simple summary will be returned directly.

## 1. Introduction

According to the World Health Organization (WHO) classification of tumors, gestational trophoblastic disease (GTD) is a group of disorders characterized by increased trophoblast proliferation and impaired expression of imprinted gene products [1,2,3,4,5].

GTD comprises complete (CHM), partial (PHM), and invasive/metastatic hydatidiform moles, as well as a spectrum of four entities grouped under the term malignant gestational trophoblastic neoplasia (GTNs), including gestational choriocarcinoma, placental site trophoblastic tumor (PSTT), epithelioid trophoblastic tumor (ETT), and mixed trophoblastic tumor (MTT). GTD can be complicated by bleeding, trophoblastic pulmonary embolism, as well as preeclampsia or hyperthyroidism [1,5,6,7]. The latter has been reported in 5% of molar pregnancies [8]. Hershman and Higgins were the first to report severe hyperthyroidism associated with a hydatidiform mole in 1971 [8].

The main features associated with high risk of hyperthyroidism are uterine fundal height > 16 cm, theca lutein cysts > 6 cm, and human chorionic gonadotropin (hCG) levels > 400,000 IU/L at presentation [9]. Hyperthyroidism is accompanied by tachycardia, heat intolerance, losing weight, tremor, nervousness, and palpitations. Sometimes, it can be life threatening and requires immediate treatment if a thyroid storm occurs. Although the latter is rare, it can be fatal in 15% of cases. Unfortunately, a primary concern of hyperthyroidism is its underdiagnosis, especially at the beginning of the disease, so there is no effective early diagnosis of hyperthyroidism in patients with gestational trophoblastic disease to prevent severe complications [5,10,11].

The specific mechanisms underlying the relationship between trophoblastic tissue and hyperthyroidism in gestational trophoblastic disease are associated with increased trophoblast proliferation, which can stimulate thyroid function via increasing the half-life of thyroxine-binding globulin. In addition, the increased hCG demonstrates cross-reactivity with the thyroid-stimulating hormone due to similar α-subunits. Moreover, basic isoforms of hCG may facilitate thyrotropic activity [3,5,7,8].

Recent developments in the various medical branches have led to a renewed interest in the pathophysiology of hyperthyroidism during GTD. Previous studies have reported that the main thyroid stimulating agent is considered to be trophoblastic tissue [5,11]. However, reviews of the exact mechanisms of this complication are insufficient and scant, and most studies in this field have only focused on particular case reports. We performed the first systematic literature review of histologically confirmed GTN cases (choriocarcinoma, ETT, PSTT, MTT) associated with hyperthyroidism/thyrotoxicosis. Moreover, we tried to clarify several pathways of hyperthyroidism in GTD.

## 2. Gestational Trophoblastic Disease: Overview

### 2.1. Molar Pregnancies

Molar pregnancies occur in about 1–3: 1000 cases in developed countries [1,4,12].

PHMs and CHMs are abnormal gestations with trophoblastic proliferation and hydrops of villi, with (PHM) or without (CHM) embryonic development, histologically evident as fetal villus vessels containing red blood cells. However, the morphological features are not completely specific, and the histological features of PHM significantly overlap with those of CHM, hydropic abortion, trisomy syndromes, and other abnormalities of chromosomes, placental mesenchymal dysplasia, or twin gestations of CHM + normal fetus [1,12,13].

Most CHMs are sporadic with a de novo absence of the maternal genome (only maternal mitochondrial DNA is present) and an overexpression of a paternal-only genome, frequently diploid (80–90% monospermic/homozygous, 10–20% dispermic/heterozygous) and rarely tetraploid [12,13,14]. Loss of maternal DNA may also occur during post-zygotic diploidization of a triploid conceptus, as suggested by experimental studies revealing that androgenetic blastomeres can arise owing to whole genome segregation errors in both human and bovine embryos [1,12,15,16].

In addition, less than 3% of cases are familiar recurrent biparental CHMs, typically due to *NLRP7* (18q13.4) or *KHDC3L* (6q13) gene mutations [1,17,18,19,20,21,22,23].

P57 is a cyclin-dependent kinase inhibitor encoded by the *CDKN1C* gene (11p15.5), which is paternally imprinted and maternally expressed (lacking in CHMs). According to the WHO, the immunohistochemical marker p57 does not stain the nuclei of cytotrophoblast and villous stromal cells in sporadic and familiar CHMs, while PHMs, hydropic abortions, and non-molar early gestations show retained p57 positivity [1,24]. Decidua and intermediate trophoblastic cells are positive in all of these entities and in normal pregnancy, serving as an internal positive control. Especially if combined with short tandem repeat (STR)-DNA genotyping, p57 immunohistochemical evaluation may efficiently overcome morphology-based suboptimal diagnostic reproducibility. However, discrepancies between p57, morphology, and genetic results have been reported; indeed, aberrant retained expression has rarely been reported in CHMs, as well as loss of p57 in expression in PHMs or non-molar abortus. Molecular genotyping may not elucidate all of the possible mechanisms for loss of p57 expression [1,24,25,26,27,28,29,30,31].

Short tandem repeat DNA genotyping provides a precise diagnosis by detecting a diandric triploidy in PHMs or absence of maternal genetic contribution (androgenic-only genome) in CHMs [1,32].

hCG is a hormone produced primarily by syncytiotrophoblastic cells (during normal or molar pregnancies, as well as by GTNs or tumors with syncytiotrophoblastic differentiation), while smaller amounts of hCG are also produced in the pituitary gland, liver, and colon [12]. Curettage or hysterectomy is usually curative for hydatiform moles, but serial monitoring of hCG is required for molar pregnancies, as the risks of persistent GTD (mainly invasive hydatiform mole, IHM) and choriocarcinoma are 0.5–5% and <0.5% after PHM, respectively, while they account for about 20% and 3% after CHM, respectively. Asian and African race, as well as advanced maternal age, are other risk factors for the development of choriocarcinoma [1].

IHM is a mole (usually a CHM) retaining the villous histology but invading the myometrium and/or uterine vessels. Metastatic hydatiform moles (MHMs) may present as lesions containing abnormal molar chorionic villi outside of the uterine cavity (mainly the pelvis and the vagina). In the absence of severe hemorrhages, chemotherapy is highly effective (>80% cure rate, depending on the extent of disease) [1,33].

### 2.2. Gestational Trophoblastic Neoplasms

Choriocarcinoma is a rare and aggressive trophoblastic neoplasm that can develop from pregnancies (gestational choriocarcinoma; incidence of 1–9 per 40,000 pregnancies, higher in Asia and Africa) as a pure or mixed germ cell tumor (non-gestational choriocarcinoma) or as a choriocarcinomatous differentiation of an epithelial tumor either in women or men. Moreover, some cases can present as MTTs with associated PSTT and/or ETT components [1,12,34,35,36,37,38]. Rarely, choriocarcinomas can arise from ectopic pregnancies, cesarean scars, or extrauterine sites (ovary, vulva, etc.). The majority of choriocarcinomas synchronously arise from or are preceded by molar pregnancies (especially CHMs, >50%), spontaneous or induced abortion (25%), normal pregnancy (22.5%), or ectopic pregnancies. The CHM can occasionally be occult and associated with a non-molar pregnancy, generating a dispermic twin pregnancy. The risk of developing choriocarcinoma is less than 0.005% after a normal pregnancy (about 1/1000 less than following a CHM) [1,39,40,41,42,43,44,45,46,47,48,49,50,51,52,53,54,55,56,57,58,59].

The management of patients with choriocarcinoma is based on the International Federation of Gynecology and Obstetrics (FIGO) staging classification (stage I: tumor confined to uterus; II: extrauterine extension limited to adnexa, vagina, and/or broad ligament; III: pulmonary involvement; IV: involvement of other sites) and the WHO prognostic score (Table 1) [1,33].

Chemotherapy is the treatment of choice, yielding an excellent response (about 86–100% cure rate) depending on tumor stage and prognostic score. In patients who are not responding to chemotherapy, the 5-year survival rate is lower (about 43%) [1,12,33,60,61,62,63,64].

Hysterectomy can decrease the number of cycles of chemotherapy needed to achieve remission in low-risk patients who do not want to preserve fertility and can be considered in uterine-confined, chemoresistant disease or in uncontrollable bleeding [65].

As mentioned, choriocarcinomas can present as mixed trophoblastic tumors in association with PSTT and/or ETT areas. PSTT and ETT are rare malignant intermediate trophoblastic tumors of implantation site type (PSTT) or chorionic type (ETT), respectively. These entities seem even rarer than choriocarcinoma.

Histologically, ETT is a destructive nodular proliferation of relatively uniform tumor cells arranged in nests or cords with characteristic eosinophilic hyaline-like material in the center of the tumor nests. Moderate nuclear atypia, areas of necrosis, variable (but frequently increased) mitotic index, a Ki-67 index of >10% (on immunohistochemistry), and high immunohistochemical expression of PD-L1 are usually found. PSTT is histologically composed of infiltrative aggregates or sheets of large polyhedral, usually mononuclear cells with abundant amphophilic cytoplasm and pronounced pleomorphism of the sometimes convoluted nuclei [66,67,68,69,70].

According to the NCCN guidelines, the prognostic score is not valid for intermediate trophoblastic tumors. Poor prognostic factors in PSTT include tumor cells with a clear cytoplasm, large tumor size, >5 mitoses per 10 high-power fields, deep myometrial invasion, extensive coagulative necrosis, lymphovascular invasion, interval since last pregnancy > 2 years, and age > 40 years [33,71].

## 3. Histologically Confirmed Gestational Trophoblastic Neoplasia Associated with Abnormal Thyroid Function: Systematic Review of the Literature

### 3.1. Methods for Our Systematic Literature Review

**Figure 1:**
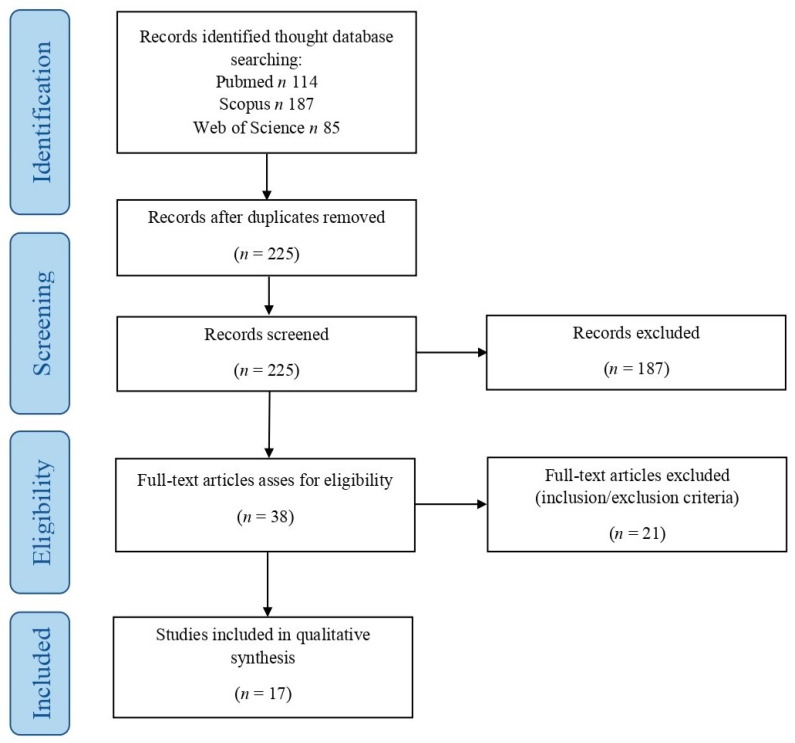
Systematic literature review: PRISMA flowchart.

We performed a systematic literature review according to the “Preferred Reporting Items for Systematic Reviews and Meta-Analyses” (PRISMA) guidelines (http://www.prisma-statement.org/, accessed on 2 February 2025) by searching for patients with histologically confirmed GTN and showing thyrotoxicosis or abnormal thyroid function. This study has been registered in the PROSPERO international prospective register of systematic reviews by the National Institute for Health Research (NIHR) (protocol and registration number: PROSPERO 2025 CRD420251037318). We used a retrospective observational approach (PICO process) (Population: as abovementioned; Intervention: any; Comparison: none; Outcomes: clinical outcomes including status at last follow-up, survival, and recurrence rates) and searched for (choriocarcinoma OR choriocarcinomas OR “epithelioid trophoblastic tumor” OR “epithelioid trophoblastic tumors” OR “placental site trophoblastic tumor” OR “placental site trophoblastic tumors” OR “epithelioid trophoblastic tumour” OR “epithelioid trophoblastic tumours” OR “placental site trophoblastic tumour” OR “placental site trophoblastic tumours” OR “mixed trophoblastic tumor” OR “mixed trophoblastic tumours” OR “gestational trophoblastic neoplasm” OR “gestational trophoblastic neoplasia”) AND (hyperthyroidism OR thyrotoxicosis OR “thyroid storm”) in PubMed (all fields, 114 results; https://pubmed.ncbi.nlm.nih.gov, accessed on 2 February 2025), Scopus (Title/Abstract/Keywords, 187 results; https://www.scopus.com/home.uri, accessed on 2 February 2025), and Web of Science (all fields, 85 results; https://webofknowledge.com, accessed on 2 February 2025). No limitations were set. The bibliographic research ended on 2 February 2025 (Figure 1).

We applied the following criteria:

- Eligibility/inclusion criteria: studies describing cases of patients with histologically confirmed GTN (choriocarcinoma, ETT, PSTT, MTT) and showing thyrotoxicosis or abnormal thyroid function.
- Exclusion criteria: unclear diagnosis; GTN not histologically confirmed; non-gestational neoplasms; too aggregated or scant data.

After duplicates’ removal, two independent authors read the titles and abstracts of all of the retrieved results (*n* = 225). By applying the eligibility/inclusion and exclusion criteria, 38 articles were considered eligible; they were all obtained in full-text format, and their reference lists were also screened to search for additional relevant articles. After reading the full texts, 21 cases were excluded as the histological diagnosis was not confirmed, choriocarcinoma was non-gestational or of uncertain origin, or data were too aggregated to clearly identify the number and features of histologically confirmed GTNs associated with abnormal thyroid function. The remaining 17 articles were finally included in our study [72,73,74,75,76,77,78,79,80,81,82,83,84,85,86,87,88]. The extracted results were checked and confirmed by two other authors. Data collection was case and study related. Categorical variables were analyzed as frequencies and percentages, continuous variables by ranges and mean values. Given the limited number of cases, no further statistical analysis was performed.

### 3.2. Results of Our Systematic Literature Review

We identified a total of 32 gestational choriocarcinomas [72,73,74,75,76,77,78,79,80,81,82,83,84,85,86,87] and one PSTT patient [88], while no ETT or MTT cases were found.

The PSTT [88] was diagnosed 11 months after a normal vaginal delivery in a 29-year-old woman (G2P2) presenting with irregular genital bleeding and hyperthyroidism (increased appetite; sweating; fatigue; weight loss of 7 kg over 3 months; sinus tachycardia with a heart rate of 125/min; thyroid-stimulating hormone, TSH < 0.01 μIU/mL; FT3 24.3 pg/mL; FT4 5.3 ng/mL; and TRAb3rd 2.1 IU/L). The serum hCG and E2 levels were 117 mIU/mL and 51 pg/mL, respectively. The patient underwent methimazole administration, total laparoscopic hysterectomy, and bilateral salpingectomy. The PSTT was 3 cm in size, and FIGO stage I. Postoperative hCG levels rapidly became negative, along with thyroid normalization. Methimazole was gradually discontinued 1 year after surgery, and no evidence of disease or hyperthyroidism was found 2 years later. In addition (a case excluded from our analysis), Moore-Maxwell et al. reported a 48-year-old G2 woman with preeclampsia, hyperthyroidism, and elevated hCG. After a curettage diagnosis of CHM, the hCG levels decreased for a short time, but they rapidly increased despite methotrexate administration, and a new curettage diagnosed a PSTT. In this case, it was unclear if the hyperthyroidism symptoms persisted with disease progression [89].

Table 2 reports the clinical data of the histologically confirmed gestational choriocarcinoma cases associated with hyperthyroidism [72,73,74,75,76,77,78,79,80,81,82,83,84,85,86,87].

The age of choriocarcinoma patients ranged from 15 to 45 years (mean age: 27 years; median age: 26 years). Two cases seemed to arise from a tubal ectopic pregnancy [73,87], while one case was intraplacental [75].

Lungs were involved by metastases in most of the cases (17/32, 53%), followed by the brain (8/32, 25%), liver (6/32, 19%), kidneys (2/32, 6%), spleen (2/32, 6%), ovaries (2/32, 6%), vagina (2/32, 6%), pelvis/abdomen (2/32, 6%), and thyroid (1/32, 3%) [72,73,74,75,76,77,78,79,80,81,82,83,84,85,86,87]. In three cases (9%), the metastases were widespread, while the extent of disease was unclear in the remaining patients. Time to recurrence was available for just five cases (16%), ranging from 1 to 36 months (mean 12 months). Status at last follow-up was available for 31 cases (97%) (mean follow-up 14 years, range: 2–72 years), and 10 patients (32%) were alive with disease (AWD; follow-up time available for 2 cases, 6 and 15 months, respectively; mean follow-up 10.5 months), 6 cases (19%) showed no evidence of disease (NED, 2–18 months, mean 8.6 months), and most of the women (15 cases, 48%) died of disease (DOD) after 4–72 months (mean 17 months) [72,73,74,75,76,77,78,79,80,81,82,83,84,85,86,87].

The serum levels of hCG ranged from 10,000 to 3,058,000,000 IU/L (mean 128,957,613 IU/L). T3 and T4 hormones were both usually increased in each patient with available data, even if different range references and units of measurement were used [72,73,74,75,76,77,78,79,80,81,82,83,84,85,86,87].

When reported, at least some symptoms and/or signs of hyperthyroidism were evident with variable intensity in most of the patients [72,73,75,77,78,80,81,82,83,84], while one case showed subclinical hyperthyroidism [76]. Normalization or significant improvement of symptoms and laboratory data usually occurred within 2–3 weeks after treatment administration [72,73,74,75,76,80,81,82,83,84,85].

## 4. Pathways of Hyperthyroidism in GTD

### 4.1. Thyroxine-Binding Globulin and Hyperestrogenism

Due to the rarity of GTNs, most experimental studies investigating the pathways of hyperthyroidism in GTD actually studied molar pregnancies [90,91,92,93,94,95,96,97,98,99,100,101,102,103,104,105,106,107,108]. Hydatidiform moles could enhance thyroid function with the help of thyroxine-binding globulin (TBG), which is encoded by the *SERPINA7* gene [90,91]. It is a glycoprotein with a molecular mass of 54 kDa, and it plays an important role in thyroid hormone serum levels via binding triiodothyronine (T3) and thyroxine (T4) with high affinity but low capacity for abovementioned hormones [90,91].

In normal pregnancies, TBG levels arise in response to increased estradiol levels, reaching a plateau around 20 gestational weeks and causing a 1.5-fold elevation of total T4 and T3 hormones [60,104]. Likewise, in hydatidiform moles, trophoblast cells convert dehydroisoandrosterone sulfate (DHEAS) to estradiol, causing hyperestrogenism [92]. Then, elevated estrogen levels contribute to the increase in the complexity of oligosaccharide side-chains, which appropriately increase the number of sialic acids in the TBG molecule. Subsequently, this mechanism prevents uptake and delays TBG degradation by the liver, causing its high serum level [90,93].

The elevated TBG serum level induces a decline of T3 and T4 free forms, thereby leading to increased TSH or thyrotropin. TSH can be defined as a heterodimeric glycoprotein that consists of two (α and β) subunits. This hormone interacts with TSH receptors (TSH-R) (a subfamily of type A G protein-coupled receptors that signal through the cyclic adenomonophosphate (cAMP) and inositol phosphate pathways) on thyroid follicular cells and triggers thyroid function [94,95].

Familial gestational hyperthyroidism (FGH) is a rare disease due to TSH receptor (TSH-R) mutations (like K183R) causing TSH-R hypersensitivity to normal hCG levels during normal pregnancies or GTD, favoring the rise of hyperthyroidism symptoms. Cell line transfection studies found no differences in membrane expression of the mutated TSH-R and similar basal and TSH-stimulated cAMP levels. The K183R mutation increased the sensitivity of TSH-R for hCG (which was still 1000 times less responsive to hCG than the LH/CG receptor), but it remained unaltered for the cognate ligand TSH [60,109].

### 4.2. Human Chorionic Gonadotropin and Thyroid-Stimulating Hormone

Many researchers have reported the association between increased hCG and decreased thyroid-stimulating hormone (TSH) serum levels [7,96]. Increased hCG levels can lead to overproduction of thyroid hormones. A temporary increase in thyroid function occurs in 1.4–11% of normally pregnant women (gestational transient thyrotoxicosis), mostly when hCG levels are above 70–80,000 IU/L [88,97,98,110,111].

hCG levels and bioactivity decrease in late pregnancy, inducing gestational hyperthyroidism that is typically transient and limited to the first 3–4 gestational months; however, some women are symptomatic, presenting with hyperemesis, which can also be due to marked hCG-induced increase in estradiol levels [108].

Whilst in normal pregnancy, high levels of T3 and T likely contribute to hyperemesis gravidarum, the clinical manifestation of hyperthyroidism is more frequent in hydatidiform moles and choriocarcinoma [90]. For instance, previous studies have indicated that in molar pregnancies, thyroid function was increased in 20% to 64% of cases [7], while hCG levels in PSTT/ETT patients are not as high as in normal pregnancies or other GTD entities [88].

hCG-induced hyperthyroidism was reported in gestational choriocarcinomas as well as in rare paraneoplastic syndrome due to hCG-secreting germ cell tumors in both women and men. Indeed, about 40–60% of testicular non-gestational choriocarcinomas were associated with elevated hCG titers, but only a minority of patients (3.5%) developed paraneoplastic hyperthyroidism [86,99,100,101,102].

#### 4.2.1. Cross-Reactivity via Identical α-Subunit

**Figure 2:**
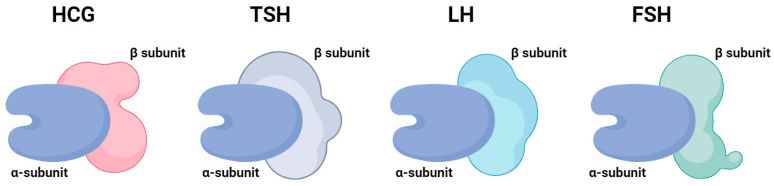
Structure of gonadotropins (glycoprotein hormones) consisting of α and β subunits. The α subunit is identical for all hormones, while the β subunit is unique and responsible for biological specificity. hCG—human chorionic gonadotropin, TSH—thyroid-stimulating hormone, LH—luteinizing hormone, FSH—follicle-stimulating hormone (previously unpublished original photo).

hCG can be defined as a glycoprotein hormone that is synthesized predominantly in the syncytiotrophoblast. hCG molecules may act as a thyrotropin and weakly activate TSH receptors, as hCG and TSH share the same subunits, which can lead to cross-stimulation of the TSH receptor [60]. In particular, hCG is composed of two subunits:

- The α subunit is coded on chromosome 6 and consists of 92 amino acid residues with two nitrogen-linked oligosaccharide side-chains. It is identical to TSH, luteinizing hormone (LH), and follicle-stimulating hormone (FSH), so it has conclusively been shown that a cross-reactivity between these hormones could exist [90,103].
- The β subunit determines specificity for each of the abovementioned glycoprotein hormones, encoded in a cluster on chromosome 19 (Figure 2) [90].

So, high doses of hCG (like during the first trimester of gestation and especially in twin pregnancies) cross-react with the TSH receptor, increasing the secretion of T4 and T3, with subsequent suppression of TSH secretion and a decrease in TSH levels. Indeed, at 6–20 gestational weeks, free T4 levels increase linearly with the rising hCG levels, while the hCG and TSH levels seem inversely correlated; however, clinically evident hyperthyroidism may occur in a subset of pregnant women [81,111,112,113,114,115,116]. Likewise, LH has intrinsic thyroid stimulating activity (lower than hCG) [60].

GTD causes marked elevation of serum thyroid hormone (T4, T3, and free T4) concentrations in some patients, but clinical thyrotoxicosis is usually absent. According to some studies, the level of thyroid stimulation and the severity of clinical hyperthyroidism may be directly proportional to the hCG concentration, causing clinical evidence of thyrotoxicosis for serum hCG levels greater than 100,000 IU/L [39]. In our review, when data were available, all except two cases [78,81] showed a greater hCG level. However, the two cases with lower levels also seemed to show some hyperthyroidism symptoms. Thyroid stimulation depends on the amplitude and duration of the hCG peak in normal pregnancies; if choriocarcinoma resists drug treatments or recurs with a new rise in the hCG levels, it may result in persistence or relapse of hyperthyroidism [99].

In some GTD cases, the serum hCG level could be subject to the high-dose Hook effect when using routine assays, thus resulting in falsely low hCG measurement and creating a clinical challenge favoring misdiagnosis. Serum dilution before the immunometric method may help in obtaining true values [60,117,118,119].

#### 4.2.2. Thyrotropic Activity of the hCG Isoforms

A marked increase of serum hCG levels does not necessarily cause hyperthyroidism, and this could be explained by the existence of hCG variants with diverse potency and thyrotrophic activity [90,104,105]. Less likely, a TSH-R gene polymorphism (like in FGH) could also increase the thyroid receptor’s sensitivity to hCG [90,104,105].

There are several published studies describing the role of the number and structure of the oligosaccharide side-chain in the biologic activity and half-life of glycoprotein hormones, including hCG. During pregnancy, multiple hCG forms circulate in the blood and urine, including the intact hormone and each of the free subunits. hCG is primarily catabolized by the liver, but ~20% is excreted in the urine; in particular, the β subunit is degraded in the kidney to make a core fragment that is measurable by urine hCG tests [39,60,90,104,105,106].

hCG thyrotropic activity seems to be influenced by hCG metabolism. The half-life is 24 h for native acidic isoforms of hCG (the pH is 3.8) due to the high content of sialic acid [7,60,90,107]. Compared to normal hCG, the desialylated variants more effectively inhibited TSH-binding and TSH-induced adenylyl cyclase stimulation in some experimental studies. hCG also increased iodide uptake in cultured FRTL-5 cells, inducing a dose-related elevation of adenylyl cyclase activity and thymidine uptake [60].

It has been demonstrated that deglycosylation or/and desialylation result in enhancing the thyrotrophic potency of hCG, as the decrease of sialic acids facilitates the binding of basic hCG isoforms with TSH-R [67]. It has been also suggested that basic isoforms with lower sialic acid content have greater efficiency in activating the TSH-R and its cAMP-pathways, as well as a high bioactivity/immunoactivity ratio in CHO cells expressing human TSH-R; mostly basic isoforms of hCG with more thyrotropic activity were revealed in cases with hydatidiform moles. However, basic isoforms seemed to have shorter half-lives [39,103,105,108,120,121,122,123].

In view of all that has been mentioned so far, one may suppose that basic isoforms of hCG with lower content of sialic acids may be responsible for hyperthyroidism during GTD, even if studies concerning the rare GTD histotypes are scant [7,90,105,107].

#### 4.2.3. Chorionic Thyrotropin

Few studies have linked hyperthyroidism to human chorionic thyrotropin (HCT). It was demonstrated that the latter is produced by the placenta. A series of trials observed an increased level of HCT in molar pregnancy in comparison with normal pregnancy [105,124,125]. Unfortunately, to the best of our knowledge, nothing was reported in the more recent literature on this topic.

## 5. Discussion

### 5.1. Hyperthyroidism in GTD: Complications and Therapeutic Considerations

Thyrotoxicosis can be due to different causes, such as Graves’ disease (TSH-R stimulating antibodies), toxic adenoma, multinodular goiter or thyroid carcinoma (somatic gain-of-function TSH-R mutations), sporadic or familial non-autoimmune hyperthyroidism (germline gain-of-function TSH-R mutations), TSH-secreting pituitary adenoma (increased stimulation by inappropriate TSH secretion), hCG-induced gestational hyperthyroidism, familial hypersensitivity to hCG (TSH-R mutation with increased sensitivity to hCG), GTD, struma ovarii (autonomous function of thyroid tissue in ovarian teratoma; about 8% of cases with throtoxicosis), iodine-induced hyperthyroidism (increased synthesis of thyroid hormone in autonomously functioning thyroid tissue exposed to iodine excess), or iatrogenic exogenous administration of thyroid hormones (thyroxicosis factitia). Moreover, subacute/silent or drug-induced thyroiditis may cause thyrotoxicosis due to the release of stored thyroid hormones [60,72,73,74,75,76,77,78,79,80,81,82,83,84,85,86,87,88,109,126,127,128,129,130,131,132,133,134,135].

Hyperthyroidism can range from asymptomatic to thyroid storm [4]. Clinical features of hyperthyroidism associated with GTD include fatigue, weight loss, muscle weakness, tremor, increased metabolic rate, heat intolerance, accelerated intestinal transit, polyphagia, tachycardia, and minimal thyroid enlargement. It is interesting to note that ophthalmopathy was not observed. Hyperreactive reflexes and the manifestation of cardiomyopathy are possible [9,136].

Thyroid storm and heart failure are the most serious consequences of hyperthyroidism, leading to death in about 10–30% of cases [137,138].

Symptoms of thyroid storm include severe agitation, delirium, unconsciousness, hyperthermia, hyperpyrexia, diarrhea, severe dehydration, tachypnea, tachycardia, atrial fibrillation, and hemodynamic instability, leading to pulmonary edema associated with heart failure [136,137,138,139,140].

However, GTD patients usually present with subclinical hyperthyroidism or mild hyperthyroid symptoms, such as tachycardia and anxiety; the early signs of thyrotoxicosis could be misattributed to infections, other inflammatory etiologies, or worsening of primary malignancy. A low threshold of suspicion for clinically relevant hyperthyroidism should be kept. Once paraneoplastic hCG-induced thyrotoxicosis is identified, timely oncological and medical therapy should be administered; multimodal treatment may be required to achieve a biochemical euthyroid state. Initial mild hyperthyroidism may develop into a thyroid storm in response to further stress; imaging studies using iodinated contrast agents should be limited or excluded to avoid potential worsening of underlying thyrotoxicosis. Paraneoplastic hCG-induced thyrotoxicosis should only be diagnosed if primary hyperplasia and tumor development of the thyroid gland are absent. Prophylactic treatment of overactive thyroid may be required [39].

The clinical–pathologic features and treatment of some previously reported cases of hyperthyroidism in molar pregnancy are summarized in Table 3 [5,140,141,142,143,144,145,146,147,148,149,150,151].

The progression from mild hyperthyroidism to thyroid storm in patients with GTD is a rare but life-threatening complication. The effectiveness of current therapeutic interventions in preventing this progression depends on early recognition, aggressive management of hyperthyroidism, and definitive treatment of the underlying GTD. Hyperthyroidism in GTD is caused by excessive hCG stimulation of the thyroid gland (due to its structural similarity to TSH) [140,141,142,143,144,145,146,147,148,149,150,151].

Close monitoring of thyroid function (FT4, FT3, TSH) in high-risk GTD patients (e.g., those with very high hCG levels, molar pregnancies, or choriocarcinoma) is critical.

Thyrotoxic moles are treated through surgical removal of the neoplastic tissue (curettage or hysterectomy). Chemotherapy with methotrexate may help to attenuate the thyrotoxic effects of moles and is the first choice for a confirmed GTN [33,136]. The serum levels of thyroid hormones and hCG levels usually normalize rapidly after removal of the mole or effective chemotherapeutic treatment of GTNs, so there is no long-term effect of hyperthyroidism on patients after treatment of gestational trophoblastic disease.

Symptomatic and antithyroid management may be required while response to oncologic therapy is achieved. Thyroid hormone synthesis inhibitors, such as methimazole or propylthiouracil, are also used as antithyroid medications in thyrotoxic GTD to regulate the excessive production of thyroid hormones. In addition, the peripheral conversion of T4 into the more potent hormone T3 can be inhibited by corticosteroid drugs [139,140].

Depending on the severity of hyperthyroidism, thyroid medications and β-adrenergic blockers may be used to reverse the metabolic and cardiovascular features. Cooling blankets, fluid resuscitation aimed at rehydration with glucose and electrolytes, oxygen therapy, iodine supplements, and B-complex multivitamins are some of the treatment options for thyroid storm [101,105,136,137,138,139,140,141].

The presence of hyperthyroidism in patients with GTD can influence both prognosis and treatment outcomes, primarily due to its association with bulky disease, metastasis, and potential life-threatening complications like thyroid storm with surgical risks. It is suggested that hyperthyroidism is a marker of disease severity rather than an independent prognostic factor, but its presence often indicates worse progression-free and overall survival if not managed aggressively [140,141,142,143,144,145,146].

The underdiagnosis of hyperthyroidism in patients with choriocarcinoma can have severe clinical consequences, affecting both short-term management and long-term survival. It can lead to inadequate risk stratification (missed high-risk FIGO/WHO score adjustment) and suboptimal chemotherapy selection (e.g., using single-agent methotrexate instead of multi-agent EMA-CO for true high-risk cases) [147,148,149,150,151].

### 5.2. Hyperthyroidism in GTD: Guidelines and Study Limitations

Limits to evaluating and defining the accurate clinical and prognostic features of the different GTD entities, as well as the effects of treatments, include the following considerations:

1. Most studies grouped data of the various entities (moles, choriocarcinomas, ETT, PSTT) or were focused on the more common form of GTD (hydatidiform moles) [1,152,153,154,155,156,157]. Moreover, in some cases, the origin of the choriocarcinoma or its gestational nature was not clear, such as in ovarian cases that can be either non-gestational or due to an ectopic pregnancy [37,158,159,160,161,162,163,164,165,166,167,168].
2. On gross examination, intraplacental areas of choriocarcinomas may be misinterpreted as hemorrhagic areas or vascular lesions and may not be sampled. Indeed, choriocarcinomas produce placental and epidermal growth factors causing neo-angiogenesis, resulting in hypervascular and hemorrhagic lesions [1,169,170,171,172].
3. Despite potential causes of underdiagnosis, choriocarcinoma is still rare, although it represents the most common form of GTN (compared to ETT and PSTT) [1,12,33,39].
4. In most cases, the diagnosis of choriocarcinoma is retrospective, as the tumor is asymptomatic after the initial pregnancy and may present as a metastatic disease. Moreover, not all women routinely undergo βHCG monitoring and placental histological exam. So, histological data of the antecedent background may not be available [1,12,39,40,173,174].
5. Moreover, the accurate diagnosis of each entity in the spectrum of GTD is histopathological (with the potential ai of immunohistochemistry and genetic/molecular analysis), but histopathological confirmation was not always performed in the literature and is not mandatory according to clinical guidelines [33,96,175,176,177,178,179,180]. Indeed, patients can be diagnosed with persistent/metastatic GTN based on clinical, laboratory (serum levels of βHCG), and imaging findings that can also be sufficient to decide the type of treatment [174]. Unfortunately, this approach excludes the possibility of a proper definition of the histological entity and its features, and it is an obstacle to obtaining data and collectible histopathological material for research purposes of a rare neoplasm, even if it spares invasive biopsy for the patient. Indeed, although choriocarcinoma has a propensity for rapid progression and widespread metastases, metastatic GTD can also be due to the other GTN entities, such as ETT/PSTT, that can metastasize or recur in 25–30% of cases [1]. Moreover, invasive and metastatic moles were also reported in the literature [1,181,182,183,184,185,186,187].
6. In addition, when a biopsy is performed, histopathological tumor heterogeneity (such as mixed GTNs or choriocarcinomas synchronously arising from moles) and tumor progression to choriocarcinomas or other GTN can cause sampling and classification biases, missing some tumor components [1,12,39,66,67,68,69,70,184].

To our knowledge, we performed the first systematic literature review of histologically confirmed GTN cases associated with hyperthyroidism/thyrotoxicosis. We feel that point of strengths of our work are the focus on only certain histologically confirmed cases and the discussion of the different pathways of hyperthyroidism in GTD.

Limits of our study may include the following: (1) some GTN cases grouped as GTD may have not been found by our search terms; (2) cases without a histological diagnosis were excluded according to our search approach, but we cannot exclude that non-biopsied cases were GTN, although we do not know the exact GTN histotype; and (3) data were scant or unavailable in some cases, including with regard to hyperthyroidism pathways (compared to molar pregnancies).

## 6. Conclusions

Hyperthyroidism in GTD is a rare and frequently subclinical event. However, symptoms may develop and may be underdiagnosed if subtle. Thyroid hormone screening should be advised in GTD patients in order to prevent clinical symptoms and/or treat them early and properly with adequate therapy in order to avoid severe consequences; indeed, after treatment, the symptoms usually regress after 2–3 weeks. Hyperthyroidism during GTD has been attributed to multiple pathways, e.g., via the stimulatory effect of hCG on the TSH receptor and high thyrotropic activity of basic hCG isoforms with less sialic acid in cases of chemoresistance or GTN progression/recurrence (with elevation of hCG levels). A multidisciplinary approach is necessary to exclude other causes of hyperthyroidism and for the management of patients with hyperthyroidism secondary to GTD. Further studies on histologically confirmed cases may provide more detailed information on different GTN subtypes.

## Figures and Tables

**Table 1:** The WHO risk scoring system.

Parameter	Points			
	0	1	2	4
Age (years)	<40	≥40	-	-
Antecedent pregnancy	Mole	Abortion	Term	-
Interval from index pregnancy (months)	<4	4–6	7–12	>12
Pretreatment hCG (mIU/mL)	<10^3^	>10^3^–10^4^	>10^4^ to 10^5^	>10^5^
Largest tumor size, including uterus (cm)	-	3–4	≥5	-
Site of metastases	Lung	Spleen, kidney	Gastrointestinal	Brain, liver
Number of metastases	-	1–4	5–8	>8
Previous failed chemotherapy (chemoresistance)	-	-	Single drug	≥2 drugs

Low risk: ≤6; high risk: ≥7.

**Table 2:** Clinical cases of hyperthyroidism in histologically confirmed gestational choriocarcinomas.

Case	Age	Beta-hCG	Metastases	Treatment	Follow-Up
Gupta et al., 2024 [72]	38	663,200 IU/L (serum)	Lungs, brain	PTU, esmolol, PRO/atenolol, MET, MTX/ETO, leucovirin, ACT, cyclophosphamide, total hysterectomy	NED, 6 months
Saleem et al., 2021 [73]	30	3,058,000,000 IU/L (serum)	Lungs, liver, spleen, brain, kidneys	MTX/ETO, CAR/PRO, cholestyramine, Lugol’s iodine, antiepileptic drugs	AWD
Tong et al., 2017 [74]	31	>200,000 IU/L (serum)	Lungs	Lugol’s iodine, CAR/PRO, uterine evacuation	AWD
Subang et al., 2016 [75]	34	1,433,740 IU/L (serum)	Lungs	PTU/MET; after delivery, MTX/ACT/ETO Methasone, leucovirin	DOD
Meister et al., 2005 [76]	26	2,564,768 IU/L (serum)	Lungs	MTX/ACT/ETO/folic acid, PRO	NED, 6 mo
Ismail et al., 2000 [77]	28	125,000 IU/L (serum)	Lungs	NR	NR
O’Reilly et al., 1993: case 1 [78]	40	>10,000 IU/L (serum)	Lungs, right ovary	No	DOD
Soutter et al., 1981: case 1 [80]	45	750,000 IU/L (serum)	Lungs	Hydroxyurea, MTX, vincristine, ACT, cyclophosphamide, folinic acid, iodide therapy	AWD
Soutter et al., 1981: case 2 [80]	26	7000 IU/L, then 480,000 IU/L (serum)	Lungs, brain (after 6 mo)	MTX, folinic acid, lost at follow-up, MTX/besamethasone/folinic acid, PRO, chlorpromazine, iodide therapy	AWD, 15 mo
Norman et al., 1981: case 1 [79]	NR	9 × 10^6^ IU/L (serum)	NR	NR	AWD
Norman et al., 1981: case 2 [79]	NR	4 × 10^6^ IU/L (serum)	NR	NR	DOD
Norman et al., 1981: case 3 [79]	NR	1.37 × 10^6^ IU/L (serum)	NR	NR	DOD
Norman et al., 1981: case 4 [79]	NR	0.75 × 10^6^ IU/L (serum)	NR	NR	AWD
Norman et al., 1981: case 5 [79]	NR	0.48 × 10^6^ IU/L (serum)	NR	NR	AWD
Norman et al., 1981: case 6 [79]	NR	0.38 × 10^6^ IU/L (serum)	Widespread	Chemotherapy	DOD
Norman et al., 1981: case 7 [79]	NR	0.17 × 10^6^ IU/L (serum)	Widespread	Chemotherapy	DOD
Norman et al., 1981: case 8 [79]	NR	0.1 × 10^6^ IU/L (serum)	NR	NR	AWD
Nisula et al., 1980: case 1 [81]	26	14,000 IU/L (serum)	Lungs (3 and 6 years after hyd mol), liver (6 years)	Chemotherapy, PRO, PTU, 131-I uptake	DOD, 6 years
Nisula et al., 1980: case 2 [81]	19	3,220,000 IU/L (serum)	Lungs, brain	ACT, brain radiotherapy, thyrotropin-releasing factor	NED, 18 mo
Anderson et al., 1978 [82]	26	>500,000 IU/L (serum)	Lungs, liver, brain, thyroid	MTX, brain radiotherapy, ACT, hepatic wedge resection	DOD, 8 mo
Cave et al., 1976 [83]	15	9,490,000 IU/L (serum), 6,400,000–12,800,000 IU/mL (urine)	Lungs, brain, abdomen, ovaries, kidneys, liver	MTX, ACT, chlorambucil, brain radiotherapy; PRO, PTU, iodine	DOD, 4 mo
Morley et al., 1976: case 1 [84]	33	134,000 IU/L (serum), 350 IU/mL/24 h (urine)	Vagina	MTX, ACT, hysterectomy	NED, 7 mo
Morley et al., 1976: case 2 [84]	19	185,000 IU/L (serum), 360 IU/mL/24 h (urine)	Lungs, brain (3 mo)	MTX, ACT	AWD, 6 mo
Morley et al., 1976: case 3 [84]	28	963,000 IU/L (serum), 17.5 IU/mL/24 h (urine)	Vagina, lungs (15 mo)	Hysterectomy, MTX, 6-mercaptopurine, ACT, PRO, carbimazol, radiotherapy (pelvis, vagina)	DOD, 20 mo
Cohen et al., 1969 [85]	18	204,800 IU/day (urine)	Lungs	MTX, ACT, MET, subtotal hysterectomy	AWD
Odell et al., 1962: case 2 [86]	32	2 × 10^6^ mouse units/24 h (urine)	Widespread	MTX, ACT, PTU, Lugol’s 10 days terminally	DOD, 8 mo
Odell et al., 1962: case 3 [86]	23	5 × 10^6^ mouse units/24 h (urine)	Widespread (also liver and spleen)	MTX, ACT, chlorambucil, leurocristine	DOD, 8 mo
Odell et al., 1962: case 4 [86]	22	2 × 10^6^ mouse units/24 h (urine)	Widespread	MTX, ACT, hysterectomy	NED, 2 mo
Odell et al., 1962: case 5 [86]	29	5 × 10^6^ mouse units/24 h (urine)	Widespread	MTX	NED, 10 mo
Odell et al., 1962: case 6 [86]	26	2 × 10^6^ mouse units/24 h (urine)	Widespread	MTX, ACT, leurocristine	DOD, 14 mo
Odell et al., 1962: case 7 [86]	22	5–10 × 10^6^ mouse units/24 h (urine)	Widespread	MTX, nistrogen mustard, deoxynorleucine, Tapazole, Lugol’s 10 days terminally	DOD, 15 mo
Myers W.P.L, 1961: case 5 [87]	34	6–8 million IU (urine)	Lungs, liver, pelvis (1 mo), brain (6 mo)	Hysterectomy + bilateral salpingectomy, amethopterin	DOD, 6 mo

ACT: actynomycin; AWD: alive with disease; CAR: carbimazole; DOD: dead of disease; ETO: etoposide; MET: methimazole; mo: months; MTX: methotrexate; NED: no evidence of disease; NR: not reported; PRO: propranolol; PTU: propylthiouracil.

**Table 3:** Clinical cases of hyperthyroidism in molar pregnancy.

Authors	Year	Type of Mole	Age (Years)	hCG Levels	Treatment	Treatment for Hyperthyroidism
Wie JH et al. [140]	2016	PM	27	1,046,900 mIU/mL	curettage	hydralazine propranolol
Marchand L et al. [141]	2016	CM	42	762,878 IU/L	HY	N/A
Swaminathan S et al. [142]	2017	CM	20	N/A	curettage	propranolol
Virmani S et al. [143]	2017	CM	20	804,578 mIU/ml	curettage	propranolol
Simes BC et al. [144]	2018	CM	53	450,000 mIU/mL	HY + BSO	methimazole propranolol
Jayasuriya A et al. [145]	2020	CM	49	146,092,800 mIU/mL	HY + BSO	digoxin hydrocortisone propylthiouracil
Sharma S et al. [5]	2021	CM	48	1.7 million IU/L	curettage	hydrocortisone propylthiouracil
De Guzman E et al. [146]	2021	CM	49	414,600 IU/L	curettage	propranolol propylthiouracil
Wan Y et al. [147]	2021	IM	48	1286 mIU/mL	HY + BSO	nifedipine methimazole fluorouracil + actinomycin D
van den Tweel MM et al. [148]	2022	N/A	23	1.7 million IU/L	curettage	methotrexate
Da Silva Santos T et al. [149]	2022	CM	50	978,485 IU/L	HY + BSO	propranolol propylthiouracil dexamethasone
Jiménez-Labaig P et al. [150]	2022	IM	30	2,662,000 mIU/mL	curettage	corticosteroid EMA-CO
Walfish L et al. [151]	2023	CM	32	420 million IU/L	curettage	hydrocortisone propylthiouracil

PM—partial mole, CM—complete mole, EMA-CO—etoposide, methotrexate, actinomycin D/cyclophosphamide, vincristine, hCG—human chorionic gonadotropin, IM—invasive mole, HY—hysterectomy, BSO—bilateral salpingo-oophorectomy, IU—international units, N/A—not applicable.

## References

[1] WHO Classification of Tumours Editorial Board (2020). Female Genital Tumours: WHO Classification of Tumours.

[2] Wenk R.E., Peterson J., Baird M. (2022). A molecular classification of moles and its use in filiation tests. J. Forensic Sci..

[3] Hui P., Buza N., Murphy K.M., Ronnett B.M. (2017). Hydatidiform Moles: Genetic Basis and Precision Diagnosis. Annu. Rev. Pathol..

[4] Candelier J.J. (2016). The hydatidiform mole. Cell Adhes. Migr..

[5] Pereira J.V., Lim T. (2021). Hyperthyroidism in gestational trophoblastic disease—A literature review. Thyroid Res..

[6] Sharma S., Sharma S., Gandrabur L., Amin B., Rehmani R., Singh A. (2021). Molar Pregnancy Complicated by Impending Thyroid Storm. Cureus.

[7] Grzechocinska B., Gajewska M., Kedzierski M., Gajda S., Jedrzejak P., Wielgos M. (2021). Hyperthyroidism secondary to a hydatidiform mole. Ginekol. Pol..

[8] Petca A., Dimcea D.A., Dumitrașcu M.C., Șandru F., Mehedințu C., Petca R.C. (2023). Management of Hyperthyroidism during Pregnancy: A Systematic Literature Review. J. Clin. Med..

[9] Hershman J.M., Higgins H.P. (1971). Hydatidiform mole—A cause of clinical hyperthyroidism. Report of two cases with evidence that the molar tissue secreted a thyroid stimulator. N. Engl. J. Med..

[10] Ramos M.M., Maesta I., de Araújo Costa R.A., Mazeto G.M.F.S., Horowitz N.S., Elias K.M., Braga A., Berkowitz R.S. (2022). Clinical characteristics and thyroid function in complete hydatidiform mole complicated by hyperthyroidism. Gynecol. Oncol..

[11] Alexander E.K., Pearce E.N., Brent G.A., Brown R.S., Chen H., Dosiou C., Grobman W.A., Laurberg P., Lazarus J.H., Mandel S.J. (2017). 2017 Guidelines of the American Thyroid Association for the Diagnosis and Management of Thyroid Disease During Pregnancy and the Postpartum. Thyroid.

[12] Nicheperovich A., Schuster-Böckler B., Ní Leathlobhair M. (2025). Gestational trophoblastic disease: Understanding the molecular mechanisms of placental tumours. Dis. Model. Mech..

[13] Fisher R.A., Maher G.J. (2021). Genetics of gestational trophoblastic disease. Best. Pract. Res. Clin. Obstet. Gynaecol..

[14] Kajii T., Ohama K. (1977). Androgenetic origin of hydatidiform mole. Nature.

[15] De Coster T., Masset H., Tšuiko O., Catteeuw M., Zhao Y., Dierckxsens N., Aparicio A.L., Dimitriadou E., Debrock S., Peeraer K. (2022). Parental genomes segregate into distinct blastomeres during multipolar zygotic divisions leading to mixoploid and chimeric blastocysts. Genome Biol..

[16] Golubovsky M.D. (2003). Postzygotic diploidization of triploids as a source of unusual cases of mosaicism, chimerism and twinning. Hum. Reprod..

[17] Anvar Z., Jafarpour F., Jahromi B.N., Riccio A., Nasr-Esfahani M.H., Cubellis M.V. (2025). A Maternal Loss-of-Function Variant in KHDC3L Gene Causes a Range of Adverse Pregnancy Outcomes: A Case Report. Mol. Genet. Genomic Med..

[18] Gonzalez J., Popp M., Ocejo S., Abreu A., Bahmad H.F., Poppiti R. (2024). Gestational Trophoblastic Disease: Complete versus Partial Hydatidiform Moles. Diseases.

[19] Slim R., Fisher R., Milhavet F., Hemida R., Rojas S., Rittore C., Bagga R., Aguinaga M., Touitou I. (2022). Biallelic NLRP7 variants in patients with recurrent hydatidiform mole: A review and expert consensus. Hum. Mutat..

[20] Mahadevan S., Wen S., Wan Y.W., Peng H.H., Otta S., Liu Z., Iacovino M., Mahen E.M., Kyba M., Sadikovic B. (2014). NLRP7 affects trophoblast lineage differentiation, binds to overexpressed YY1 and alters CpG methylation. Hum. Mol. Genet..

[21] Gu B., Le G.H., Herrera S., Blair S.J., Meissner T.B., Strominger J.L. (2024). HLA-C expression in extravillous trophoblasts is determined by an ELF3-NLRP2/NLRP7 regulatory axis. Proc. Natl. Acad. Sci. USA.

[22] Zhang W., Chen Z., Zhang D., Zhao B., Liu L., Xie Z., Yao Y., Zheng P. (2019). KHDC3L mutation causes recurrent pregnancy loss by inducing genomic instability of human early embryonic cells. PLoS Biol..

[23] Demond H., Anvar Z., Jahromi B.N., Sparago A., Verma A., Davari M., Calzari L., Russo S., Jahromi M.A., Monk D. (2019). A KHDC3L mutation resulting in recurrent hydatidiform mole causes genome-wide DNA methylation loss in oocytes and persistent imprinting defects post-fertilisation. Genome Med..

[24] Akbarzadeh-Jahromi M., Taheri T., Aslani F.S., Safaei A., Pouraminaee F., Zare M. (2024). Diagnosis of hydatidiform moles using p57 immunohistochemistry and chromogenic insitu hybridization: A retrospective study. Int. J. Reprod. Biomed..

[25] Donzel M., Gaillot-Durand L., Joubert M., Aziza J., Beneteau C., Mauduit C., Ploteau S., Hajri T., Bolze P.A., Massardier J. (2023). Androgenetic/biparental mosaicism in a diploid mole-like conceptus: Report of a case with triple paternal contribution. Virchows Arch..

[26] Lu B., Ma Y., Shao Y., Xu E. (2022). Twin pregnancy with complete hydatidiform mole and co-existing fetus: A report of 15 cases with a clinicopathological analysis and DNA genotyping. Pathol. Res. Pract..

[27] Xing D., Miller K., Beierl K., Ronnett B.M. (2022). Loss of p57 Expression in Conceptions Other Than Complete Hydatidiform Mole: A Case Series With Emphasis on the Etiology, Genetics, and Clinical Significance. Am. J. Surg. Pathol..

[28] Murphy K.M., Carrick K., Gwin K., Rogers V., Koduru P., Ronnett B.M., Castrillon D.H. (2022). Rare Complete Hydatidiform Mole with p57 Expression in Villous Mesenchyme: Case Report and Review of Discordant p57 Expression in Hydatidiform Moles. Int. J. Gynecol. Pathol..

[29] Oranratanaphan S., Khongthip Y., Areeruk W., Triratanachat S., Tantbirojn P., Phupong V., Vongpaisarnsin K., Lertkhachonsuk R. (2020). Determination of morphologic and immunohistochemical stain (p57 ^kip2^) discrepancy of complete and partial hydatidiform mole by using microsatellite genotyping. Taiwan. J. Obstet. Gynecol..

[30] Hasanzadeh M., Sharifi N., Farazestanian M., Nazemian S.S., Madani Sani F. (2016). Immunohistochemistry Study of *P53* and *C-erbB-2* Expression in Trophoblastic Tissue and Their Predictive Values in Diagnosing Malignant Progression of Simple Molar Pregnancy. Iran. J. Cancer Prev..

[31] Santandrea G., Piana S., Valli R., Zanelli M., Gasparini E., De Leo A., Mandato V.D., Palicelli A. (2021). Immunohistochemical Biomarkers as a Surrogate of Molecular Analysis in Ovarian Carcinomas: A Review of the Literature. Diagnostics.

[32] Rozenova K.A., Buza N., Hui P. (2025). Gestational trophoblastic disease: STR genotyping for precision diagnosis. Expert Rev. Mol. Diagn..

[33] https://www.nccn.org/guidelines/guidelines-detail?category=1&id=1489.

[34] Kingdon S.J., Coleman R.E., Ellis L., Hancock B.W. (2012). Deaths from gestational trophoblastic neoplasia: Any lessons to be learned?. J. Reprod. Med..

[35] Kohorn E.I. (2014). Worldwide survey of the results of treating gestational trophoblastic disease. J. Reprod. Med..

[36] Cheung A.N., Zhang H.J., Xue W.C., Siu M.K. (2009). Pathogenesis of choriocarcinoma: Clinical, genetic and stem cell perspectives. Future Oncol..

[37] Ao X., Hu S., Tan S., Xiong W. (2024). Nongestational ovarian choriocarcinoma with bilateral teratoma: A rare case report and literature review. Medicine.

[38] Niu N., Buza N., Hui P. (2025). Mixed Gestational Trophoblastic Tumors-Challenging Clinicopathological Presentations. Int. J. Gynecol. Pathol..

[39] Hsieh T.Y., Hsu K.F., Kuo P.L., Huang S.C. (2008). Uterine choriocarcinoma accompanied by an extremely high human chorionic gonadotropin level and thyrotoxicosis. J. Obstet. Gynaecol. Res..

[40] Dai G.L., Tang F.R., Ma Y., Wang D.Q. (2024). Postpartum choriocarcinoma—A rare cause of delayed postpartum hemorrhage: Four case reports and literature review. Medicine.

[41] Geravandi M., Hajihashemi A., Adibi A., Habibi Tirtashi R. (2024). Post molar choriocarcinoma with solitary renal metastasis in the absence of primary uterine tumor: A case report and review of the literature. J. Med. Case Rep..

[42] Lin M., Chen J., Liao B., He Z., Lin S., Luo Y. (2021). When a vesicular placenta meets a live fetus: Case report of twin pregnancy with a partial hydatidiform mole. BMC Pregnancy Childbirth.

[43] Takano N., Takamura M., Mizuno Y., Mizuno Y., Tamaru S., Nakamura K., Soma H., Kajihara T. (2024). Genetic and histological analysis intraplacental choriocarcinoma: A case report. Med. Mol. Morphol..

[44] Weiss S., Amit A., Schwartz M.R., Kaplan A.L. (2001). Primary choriocarcinoma of the vulva. Int. J. Gynecol. Cancer..

[45] Palicelli A., Giaccherini L., Zanelli M., Bonasoni M.P., Gelli M.C., Bisagni A., Zanetti E., De Marco L., Torricelli F., Manzotti G. (2021). How Can We Treat Vulvar Carcinoma in Pregnancy? A Systematic Review of the Literature. Cancers.

[46] Han X., Qian X., Wan X., Chen Y., Chen L. (2024). Differential diagnosis of non-molar gestational trophoblastic neoplasia with ectopic pregnancy by clinical-pathological features. Arch. Gynecol. Obstet..

[47] Bartusevicius A., Bartuseviciene E., Maseviciene M., Sukovas A., Birbalaite I., Karpaviciute M. (2024). Heterotopic Tubal Choriocarcinoma Coexistent with a Viable Intrauterine Pregnancy: A Case Report. Medicina.

[48] Najib F.S., Bahrami S., Shiravani Z., Alavi S.M.A. (2023). Choriocarcinoma in tubal pregnancy: A case report. Clin. Case Rep..

[49] D’Agostino C., Surico D., Monga G., Palicelli A. (2019). Pregnancy-related decidualization of subcutaneous endometriosis occurring in a post-caesarean section scar: Case study and review of the literature. Pathol. Res. Pract..

[50] Adow M.T., Gebresilasie S.F., Abebe N.A. (2021). Primary Ovarian Choriocarcinoma: Rare Entity. Case Rep. Obstet. Gynecol..

[51] Gerson R.F., Lee E.Y., Gorman E. (2007). Primary extrauterine ovarian choriocarcinoma mistaken for ectopic pregnancy: Sonographic imaging findings. AJR Am. J. Roentgenol..

[52] Malik R., Verma M., Chauhan M., Sinha P. (2022). Choriocarcinoma of the Ovary Masquerading as Ectopic Pregnancy. Gynecol. Minim. Invasive Ther..

[53] Jashnani K.D., Sangoi N.N., Pophalkar M.P., Patil L.Y. (2022). Caesarean scar ectopic pregnancy masquerading as gestational trophoblastic disease. J. Postgrad. Med..

[54] Huang Y., Zhou T., Li Y., Gao X., Zhu Q., Wu M. (2023). Primary cesarean scar choriocarcinoma: A case series and literature review. Int. J. Gynaecol. Obstet..

[55] Yang C., Li J., Zhang Y., Xiong H., Sheng X. (2020). Epithelioid trophoblastic tumor coexisting with choriocarcinoma around an abdominal wall cesarean scar: A case report and review of the literature. J. Med. Case Rep..

[56] Wan X., Li J., Xie X. (2006). Extrauterine choriocarcinoma of the greater omentum after tubal pregnancy: Case report. Int. J. Gynecol. Cancer.

[57] Gromis J., Lee C.H., Beltre M., Khan F., Tenzel N., Zakashansky K., Kamath A. (2019). Cesarean section scar choriocarcinoma, an unusual entity with ultrasound, MRI and pathologic correlation. Clin. Imaging.

[58] Nasiri S., Hasani S.S., Mousavi A., Gilani M.M., Akhavan S., Vakili M.R. (2018). Placenta Site Trophoblastic Tumor Choriocarcinoma from Previous Cesarean Section Scar: Case Reports Iran. J. Med. Sci..

[59] Toal C., Garrett A.A., Kostadinov S., Boisen M. (2021). Gestational trophoblastic neoplasia presenting as an interstitial ectopic pregnancy. Gynecol. Oncol. Rep..

[60] Kopp P., Feingold K.R., Anawalt B., Blackman M.R., Boyce A., Chrousos G., Corpas E., de Herder W.W., Dhatariya K., Dungan K., Hofland J. (2000). Thyrotoxicosis of other Etiologies. [Updated 1 December 2010]. Endotext [Internet].

[61] Noal S., Joly F., Leblanc E. (2010). Prise en charge d’une tumeur trophoblastique gestationnelle [Management of gestational trophoblastic disease]. Gynecol. Obstet. Fertil..

[62] Liu Y.L., Praiss A.M., Chiang S., Devereaux K., Huang J., Rizzuto G., Al-Rawi D., Weigelt B., Jewell E., Abu-Rustum N.R. (2025). Gestational trophoblastic neoplasm: Patient outcomes and clinical pearls from a multidisciplinary referral center. Gynecol. Oncol..

[63] Wang Q., Fu J., Hu L., Fang F., Xie L., Chen H., He F., Wu T., Lawrie T.A. (2017). Prophylactic chemotherapy for hydatidiform mole to prevent gestational trophoblastic neoplasia. Cochrane Database Syst. Rev..

[64] Deleuze A., Massard C., Le Du F., You B., Lefeuvre-Plesse C., Bolze P.A., de la Motte Rouge T. (2023). Management of trophoblastic tumors: Review of evidence, current practice, and future directions. Expert Rev. Anticancer Ther..

[65] Elias K.M., Berkowitz R.S., Horowitz N.S. (2024). Surgical Management of Gestational Trophoblastic Neoplasia. Hematol. Oncol. Clin. N. Am..

[66] Wang V., Elias K.M., Berkowitz R.S., Horowitz N.S. (2024). Placental Site Trophoblastic Tumors and Epithelioid Trophoblastic Tumors. Hematol. Oncol. Clin. N. Am..

[67] Marquina G., Szewczyk G., Goffin F. (2024). The Rare of the Rarest: Placental Site Trophoblastic Tumor, Epithelioid Trophoblastic Tumor, Atypical Placental Site Nodule. Gynecol. Obstet. Investig..

[68] Kapoor R., Sharma A., Kamboj M., Pasricha S. (2023). Finding a speck of gold dust: Placental site trophoblastic tumor. Int. J. Gynecol. Cancer.

[69] Kaur B. (2024). Pathology of Gestational Trophoblastic Disease (GTD). Hematol. Oncol. Clin. N. Am..

[70] Li J., Du Z., Xu T., Li C., Ba S., Zhu H. (2024). Epithelioid trophoblastic tumor with lung metastasis: A case report and literature review. Medicine.

[71] Baergen R.N., Rutgers J.L., Young R.H., Osann K., Scully R.E. (2006). Placental site trophoblastic tumor: A study of 55 cases and review of the literature emphasizing factors of prognostic significance. Gynecol. Oncol..

[72] Gupta N., Graham L., Carpenter M., Gandhi G.Y. (2024). A Case of Metastatic Choriocarcinoma-Related Paraneoplastic Thyroid Storm. JCEM Case Rep..

[73] Saleem M., Sethi S.M., Ali A., Kiran Z. (2021). Metastatic choriocarcinoma in a young woman presenting as thyroid storm: A case report. J. Med. Case Rep..

[74] Tong C.V., Chai W.L. (2017). Choriocarcinoma as a cause of hyperthyroidism. QJM.

[75] Subang M.L.L., Konig M., Staats P.N., Lamos E.M., Munir K.M., Malek R. (2016). Third-Trimester Intraplacental Choriocarcinoma Presenting With Respiratory Failure and Hyperthyroidism. AACE Clin. Case Rep..

[76] Meister L.H., Hauck P.R., Graf H., Carvalho G.A. (2005). Hyperthyroidism due to secretion of human chorionic gonadotropin in a patient with metastatic choriocarcinoma. Arq. Bras. Endocrinol. Metabol..

[77] Ismail M., Bhat R.V. (2000). Thyrotoxicosis of a rare aetiology—Choriocarcinoma complicated by pulmonary secondaries and thyrotoxicosis. Postgrad. Med. J..

[78] O’Reilly S., Lyons D.J., Harrison M., Gaffney E., Cullen M., Clancy L. (1993). Thyrotoxicosis induced by choriocarcinoma a report of two cases. Ir. Med. J..

[79] Norman R.J., Green-Thompson R.W., Jialal I., Soutter W.P., Pillay N.L., Joubert S.M. (1981). Hyperthyroidism in gestational trophoblastic neoplasia. Clin. Endocrinol..

[80] Soutter W.P., Norman R., Green-Thompson R.W. (1981). The management of choriocarcinoma causing severe thyrotoxicosis. Two case reports. Br. J. Obstet. Gynaecol..

[81] Nisula B.C., Taliadouros G.S. (1980). Thyroid function in gestational trophoblastic neoplasia: Evidence that the thyrotropic activity of chorionic gonadotropin mediates the thyrotoxicosis of choriocarcinoma. Am. J. Obstet. Gynecol..

[82] Anderson N.R., Lokich J.J., McDermott W.V., Trey C., Falchuk K.R. (1979). Gestational choriocarcinoma and thyrotoxicosis. Cancer.

[83] Cave W.T., Dunn J.T. (1976). Choriocarcinoma with hyperthyroidism: Probable identity of the thyrotropin with human chorionic gonadotropin. Ann. Intern. Med..

[84] Morley J.E., Jacobson R.J., Melamed J., Hershman J.M. (1976). Choriocarcinoma as a cause of thyrotoxicosis. Am. J. Med..

[85] Cohen J.D., Utiger R.D. (1970). Metastatic choriocarcinoma associated with hyperthyroidism. J. Clin. Endocrinol. Metab..

[86] Odell W.D., Bates R.W., Rivlin R.S., Lipsett M.B., Hertz R. (1963). Increased thyroid function without clinical hyperthyroidism in patients with choriocarcinoma. J. Clin. Endocrinol. Metab..

[87] Myers W.P.L. (1961). An analysis of medical problems in cancer. Med. Clin. N. Am..

[88] Ishii S., Hirayama T., Saeki H., Fujino K., Terao Y., Itakura A. (2025). A case of transient hyperthyroidism induced by placental site trophoblastic tumor. J. Obstet. Gynaecol. Res..

[89] Moore-Maxwell C.A., Robboy S.J. (2004). Placental site trophoblastic tumor arising from antecedent molar pregnancy. Gynecol. Oncol..

[90] Hershman J.M. (2004). Physiological and pathological aspects of the effect of human chorionic gonadotropin on the thyroid. Best Pract. Res. Clin. Endocrinol. Metab..

[91] Fang Y., Chen H., Chen Q., Wang C., Liang L. (2021). Compound hemizygous variants in SERPINA7 gene cause thyroxine-binding globulin deficiency. Mol. Genet. Genomic Med..

[92] Kumar P., Magon N. (2012). Hormones in pregnancy. Niger. Med. J..

[93] Refetoff S., Feingold K.R., Anawalt B., Blackman M.R., Boyce A., Chrousos G., Corpas E., de Herder W.W., Dhatariya K., Dungan K., Hofland J. (2023). Thyroid Hormone Serum Transport Proteins. Endotext [Internet].

[94] Szkudlinski M.W. (2015). New Frontier in Glycoprotein Hormones and Their Receptors Structure-Function. Front. Endocrinol..

[95] Ząbczyńska M., Kozłowska K., Pocheć E. (2018). Glycosylation in the Thyroid Gland: Vital Aspects of Glycoprotein Function in Thyrocyte Physiology and Thyroid Disorders. Int. J. Mol. Sci..

[96] Walkington L., Webster J., Hancock B.W., Everard J., Coleman R.E. (2011). Hyperthyroidism and human chorionic gonadotrophin production in gestational trophoblastic disease. Br. J. Cancer.

[97] Khomphaiboonkij U., Termsarasab C. (2021). Can Pretreatment Serum Beta-hCG be Used for Predicting Thyrotoxicosis in Gestational Trophoblastic Disease?. Asian Pac. J. Cancer Prev..

[98] Yeo C.P., Khoo D.H., Eng P.H., Tan H.K., Yo S.L., Jacob E. (2001). Prevalence of gestational thyrotoxicosis in Asian women evaluated in the 8th to 14th weeks of pregnancy: Correlations with total and free beta human chorionic gonadotrophin. Clin. Endocrinol..

[99] Sotello D., Rivas A.M., Test V.J., Lado-Abeal J. (2016). Choriocarcinoma presenting with thyrotoxicosis. Bayl. Univ. Med. Cent. Proc..

[100] Voigt W., Maher G., Wolf H.-H., Schmoll H.J. (2007). Human chorionic gonadotropin-induced hyperthyroidism in germ cell cancer—A case presentation and review of the literature. Oncol. Res. Treat..

[101] Derakhshani P., Klotz T., Heidenreich A., Engelmann U. (1999). Diffuse metastasized testicular teratoma and paraneoplastic thyreotoxicosis. Case report and literature review. Urol. Int..

[102] Oosting S.F., de Haas E.C., Links T.P., De Bruin D., Sluiter W.J., De Jong I.J., Hoekstra H.J., Sleijfer D.T., Gietema J.A. (2010). Prevalence of paraneoplastic hyperthyroidism in patients with metastatic non-seminomatous germ-cell tumors. Ann. Oncol..

[103] Yoshimura M., Pekary A.E., Pang X.P., Berg L., Goodwin T.M., Hershman J.M. (1994). Thyrotropic activity of basic isoelectric forms of human chorionic gonadotropin extracted from hydatidiform mole tissues. J. Clin. Endocrinol. Metab..

[104] Glinoer D. (1997). The regulation of thyroid function in pregnancy: Pathways of endocrine adaptation from physiology to pathology. Endocr. Rev..

[105] Chivukula K.K., Toro-Tobón D., Motazedi B., Goyal R. (2021). Thyroid storm as an early presentation of hCG-producing metastatic choriocarcinoma: A case report and review of the literature. BMJ Case Rep..

[106] Betz D., Fane K. (2023). Human Chorionic Gonadotropin. StatPearls [Internet].

[107] Elliott M.M., Kardana A., Lustbader J.W., Cole L.A. (1997). Carbohydrate and peptide structure of the alpha- and beta-subunits of human chorionic gonadotropin from normal and aberrant pregnancy and choriocarcinoma. Endocrine.

[108] Yoshimura M., Hershman J.M. (1995). Thyrotropic action of human chorionic gonadotropin. Thyroid.

[109] Rodien P., Bremont C., Sanson M.L., Parma J., Van Sande J., Costagliola S., Luton J.P., Vassart G., Duprez L. (1998). Familial gestational hyperthyroidism caused by a mutant thyrotropin receptor hypersensitive to human chorionic gonadotropin. N. Engl. J. Med..

[110] Glinoer D., Lemone M. (1992). Goiter and pregnancy: A new insight into an old problem. Thyroid.

[111] Glinoer D. (1998). Thyroid hyperfunction during pregnancy. Thyroid.

[112] Goodwin T.M., Montoro M., Mestman J.H., Pekary A.E., Hershman J.M. (1992). The role of chorionic gonadotropin in transient hyperthyroidism of hyperemesis gravidarum. J. Clin. Endocrinol. Metab..

[113] Burrow G.N. (1993). Thyroid function and hyperfunction during gestation. Endocr. Rev..

[114] Glinoer D., De Nayer P., Robyn C., Lejeune B., Kinthaert J., Meuris S. (1993). Serum levels of intact human chorionic gonadotropin (HCG), its free alpha beta subunits in relation to maternal thyroid stimulation during normal pregnancy. J. Endocrinol. Investig..

[115] Glinoer D., de Nayer P., Bourdoux P., Lemone M., Robyn C., Steirteghem A.V., Kinthaert J., Lejeune B. (1990). Regulation of maternal thyroid during pregnancy. J. Clin. Endocrinol. Metab..

[116] Grun J.P., Meuris S., De Nayer P., Glinoer D. (1997). The thyrotrophic role of human chorionic gonadotrophin (hCG) in the early stages of twin (versus single) pregnancies. Clin. Endocrinol..

[117] Shearer A., Saso S., Stalder C., Jones B. (2024). Rare complications of complete hydatidiform molar pregnancy: The ‘hook effect’ and thyrotoxicosis. BMJ Case Rep..

[118] Phillipo D., Lucas S., Kalunga M.P., Inyasi E., Lebba J.P., Sudai F.M., Bizimana J.K. (2024). False-negative qualitative human chorionic gonadotropin (hCG) test result (‘hook effect’) with classical ultrasound findings of complete molar pregnancy: An uncommon case. Oxf. Med. Case Rep..

[119] Nizet A., Jeanmart P., Dewalque L., Bodson Q. (2023). Falsely low beta-hCG results in pregnant woman on Siemens Atellica: Don’t forget the “hook effect”. Clin. Chem. Lab. Med..

[120] Davies T.F., Platzer M. (1986). hCG-induced TSH receptor activation and growth acceleration in FRTL-5 thyroid cells. Endocrinology.

[121] Hershman J.M., Lee H.Y., Sugawara M., Mirell C.J., Pang X.P., Yanagisawa M., Pekary A.E. (1988). Human chorionic gonadotropin stimulates iodide uptake adenylate cyclase deoxyribonucleic acid synthesis in cultured rat thyroid cells. J. Clin. Endocrinol. Metab..

[122] Hoermann R., Amir S.M., Ingbar S.H. (1988). Evidence that partially desialylated variants of human chorionic gonadotropin (hCG) are the factors in crude hCG that inhibit the response to thyrotropin in human thyroid membranes. Endocrinology.

[123] Mann K., Schneider N., Hoermann R. (1986). Thyrotropic activity of acidic isoelectric variants of human chorionic gonadotropin from trophoblastic tumors. Endocrinology.

[124] Nakamura A. (1977). Human chorionic thyrotropin (hCT) and maternal thyroid function during pregnancy (author’s transl). Nihon Naibunpi Gakkai Zasshi.

[125] Tojo S., Mochizuki M., Kanazawa S. (1974). Comparative assay of HCG, HCT and HCS in molar pregnancy. Acta Obstet. Gynecol. Scand..

[126] Cosentino G., Lanzolla G., Comi S., Maglionico M.N., Posarelli C., Ciampa D.A., Menconi F., Rocchi R., Latrofa F., Figus M. (2025). Ablative Versus Conservative Approach for Hyperthyroidism Treatment in Patients with Graves’ Orbitopathy: A Retrospective Cohort Study. Thyroid.

[127] Zhu C., Liu T., Yu H., Chang L., Zhang X., Yao J., Zhang G., Chen Q., He Q., Liu M. (2024). Central hyperthyroidism due to an ectopic TSH-secreting pituitary tumor: A case report and literature review. Front. Endocrinol..

[128] Asaturova A., Magnaeva A., Tregubova A., Kometova V., Karamurzin Y., Martynov S., Lipatenkova Y., Adamyan L., Palicelli A. (2022). Malignant Clinical Course of “Proliferative” Ovarian Struma: Diagnostic Challenges and Treatment Pitfalls. Diagnostics.

[129] Dardik R.B., Dardik M., Westra W., Montz F.J. (1999). Malignant struma ovarii: Two case reports and a review of the literature. Gynecol. Oncol..

[130] (2008). Yassa L, Sadow P, Marqusee E: Malignant struma ovarii. Nat. Clin. Pract. Endocrinol. Metab..

[131] Dunzendorfer T., deLas Morenas A., Kalir T., Levin R.M. (1999). Struma ovarii and hyperthyroidism. Thyroid.

[132] Mesquita J.B., Biscolla R.P.M. (2024). Hyperthyroidism in thyroid carcinoma originating in struma ovarii. Endocrinol. Diabetes Metab. Case Rep..

[133] Yang B., Zhong L., Peng L., Huang T., Zhu D., Lu Y. (2023). Malignant Struma Ovarii (Papillary Carcinoma) with Hyperthyroidism: A Case Report and Literature Review. Case Rep. Oncol..

[134] Lai T.F., Liu Z. (2024). Hyperthyroidism and fulminant myocarditis in an adolescent with iodine-induced hyperthyroidism: A case report. SAGE Open Med. Case Rep..

[135] Hanna M., Sun B., Shekarappa R. (2024). Toxic Thyroid Adenoma Presenting as Apathetic Hyperthyroidism: A Case Report. Cureus.

[136] Filipescu G.A., Solomon O.A., Clim N., Milulescu A., Boiangiu A.G., Mitran M. (2017). Molar pregnancy and thyroid storm—Literature review. ARS Medica Tomitana.

[137] Akamizu T., Satoh T., Isozaki O., Suzuki A., Wakino S., Iburi T., Tsuboi K., Monden T., Kouki T., Otani H. (2012). Diagnostic criteria, clinical features, and incidence of thyroid storm based on nationwide surveys. Thyroid.

[138] Bourcier S., Coutrot M., Kimmoun A., Sonneville R., de Montmollin E., Persichini R., Schnell D., Charpentier J., Aubron C., Morawiec E. (2020). Thyroid storm in the ICU: A retrospective multicenter study. Crit. Care Med..

[139] Vadini V., Vasistha P., Shalit A., Maraka S. (2024). Thyroid storm in pregnancy: A review. Thyroid Res..

[140] Wie J.H., Kwon J.Y., Ko H.S., Lee Y., Shin J.C., Park I.Y. (2016). Thyroid storm and early-onset proteinuric hypertension caused by a partial molar pregnancy. J. Obstet. Gynaecol..

[141] Marchand L., Chabert P., Chaudesaygues E., Grasse M., Bretones S., Graeppi-Dulac J., Aupetit J.F. (2016). An unusual cause of cardiothyreosis. Gynecol. Endocrinol..

[142] Swaminathan S., James R.A., Chandran R., Joshi R. (2017). Anaesthetic Implications of Severe Hyperthyroidism Secondary to Molar Pregnancy: A Case Report and Review of Literature. Anesth. Essays Res..

[143] Virmani S., Srinivas S.B., Bhat R., Rao R., Kudva R. (2017). Transient Thyrotoxicosis in Molar Pregnancy. J. Clin. Diagn. Res..

[144] Simes B.C., Mbanaso A.A., Zapata C.A., Okoroji C.M. (2018). Hyperthyroidism in a complete molar pregnancy with a mature cystic ovarian teratoma. Thyroid Res..

[145] Jayasuriya A., Muthukuda D., Dissanayake P., Subasinghe S. (2020). Recurrent Thyroid Storm Caused by a Complete Hydatidiform Mole in a Perimenopausal Woman. Case Rep. Endocrinol..

[146] De Guzman E., Shakeel H., Jain R. (2021). Thyrotoxicosis: A rare presentation of molar pregnancy. BMJ Case Rep..

[147] Wan Y., Jiang G., Jin Y., Hao Z. (2021). Perimenopausal giant hydatidiform mole complicated with preeclampsia and hyperthyroidism: A case report and literature review. Open Med..

[148] van den Tweel M.M., van Dunné F.M., Johansson-Vidarsdóttir S. (2022). Hyperthyreoïdie en een onverwachte molazwangerschap [Hyperthyroidism and an unexpected molar pregnancy]. Ned. Tijdschr. Geneeskd..

[149] Da Silva Santos T., Santos Monteiro S., Pereira M.T., Garrido S., Leal M., Andrade C., Vilaverde J., Dores J. (2022). Severe Hyperthyroidism and Complete Hydatidiform Mole in Perimenopausal Woman: Case Report and Literature Review. Cureus.

[150] Jiménez-Labaig P., Mañe J.M., Rivero M.P., Lombardero L., Sancho A., López-Vivanco G. (2022). Just an Acute Pulmonary Edema? Paraneoplastic Thyroid Storm Due to Invasive Mole. Case Rep. Oncol..

[151] Walfish L., Gupta N., Nguyen D.B., Sherman M. (2023). Molar Pregnancy-Induced Hyperthyroidism: The Importance of Early Recognition and Timely Preoperative Management. JCEM Case Rep..

[152] Soper J.T. (2006). Gestational trophoblastic disease. Obstet. Gynecol..

[153] Desai R.K., Norman R.J., Jialal I., Joubert S.M. (1988). Spectrum of thyroid function abnormalities in gestational trophoblastic neoplasia. Clin. Endocrinol..

[154] Davies T.F., Taliadouros G.S., Catt K.J., Nisula B.C. (1979). Assessment of urinary thyrotropin-competing activity in choriocarcinoma and thyroid disease: Further evidence for human chorionic gonadotropin interacting at the thyroid cell membrane. J. Clin. Endocrinol. Metab..

[155] Kennedy R.L., Sheridan E., Darne J., Price A., Cohn M. (1990). Thyroid Function In Choriocarcinoma: Demonstration Of A Thyroid Stimulating Activity In Serum Using Frtl-5 And Human Thyroid Cells. Clin. Endocrinol..

[156] Zhong L., Song L., Yin R., Li Q., Wang D. (2023). Risk factors for gestational trophoblastic neoplasia development of singleton normal fetus with partial hydatidiform mole pregnancy: A retrospective cohort and literature review. J. Obstet. Gynaecol. Res..

[157] Kato K., Mostafa M.H., Mann K., Schindler A.E., Hoermann R. (2004). The human chorionic gonadotropin molecule from patients with trophoblastic diseases has a high thyrotropic activity but is less active in the ovary. Gynecol. Endocrinol..

[158] Vennin P., Demaille M.C., Saout J., Baranzelli M.C., Bonnière M. (1984). A propos d’un choriocarcinome de l’ovaire chez une femme en période d’activité génitale [A case of choriocarcinoma of the ovary in a woman during the period of genital activity]. LARC Med..

[159] Jöbsis J.J., van Trotsenburg A.S., Merks J.H., Kamp G.A. (2014). Kinderen met hyperthyreoïdie door verhoogd hCG [Children with hyperthyroidism due to elevated hCG levels]. Ned. Tijdschr. Geneeskd..

[160] Petit T., Maloisel F., Korganov A.S., Grunenberger F., Dufour P., Oberling F. (1995). Hyperthyroïdie et choriocarcinome: Une observation [Hyperthyroidism and choriocarcinoma: A case]. Ann. Med. Interne.

[161] Krige L.P., di Bisceglie A. (1984). Pitfalls in the diagnosis of gestational choriocarcinoma. A case report. S. Afr. Med. J..

[162] Haram K., Klykken B., Engebjerg E. (1979). Choriocarcinoma associated with thyrotoxicosis: A case report. Int. J. Gynecol. Obstet..

[163] Godeau P., Bletry O., Garin J.L., Amiel J.L., Lambolez T., Brochard C., Beaulieu J.L. (1980). Hyperthyroïdie par choriocacinome placentaire: Un cas avec revue de la littérature [Hyperthyroidism from placental choriocarcinoma: A case report and review of the literature (author’s transl)]. Ann. Med. Interne.

[164] Gafar I., Elhassan M., Elhaj A., Calvert P. (2024). Unusual Presentation of Non-Gestational Extragonadal Choriocarcinoma. Cureus.

[165] Sait H.K., Alghamdi F., Ragab Y., Aljadani S., Sait K.H. (2024). Non-gestational Choriocarcinoma of the Ovary: A Report of a Rare Case From Saudi Arabia. Cureus.

[166] Youssef A.T. (2024). Rare occurrence of ovarian choriocarcinoma: Ultrasound evaluation. J. Ultrasound.

[167] Mangla M., Palo S., Kanikaram P., Kaur H. (2024). Non-gestational choriocarcinoma: Unraveling the similarities and distinctions from its gestational counterpart. Int. J. Gynecol. Cancer.

[168] De Leo A., Santini D., Ceccarelli C., Santandrea G., Palicelli A., Acquaviva G., Chiarucci F., Rosini F., Ravegnini G., Pession A. (2021). What Is New on Ovarian Carcinoma: Integrated Morphologic and Molecular Analysis Following the New 2020 World Health Organization Classification of Female Genital Tumors. Diagnostics.

[169] Aonahata M., Masuzawa Y., Tsutsui Y. (1998). A case of intraplacental choriocarcinoma associated with placental hemangioma. Pathol. Int..

[170] Pérez García G.E., Sierra Avendaño J.A., Rangel Navia E., Fuentes Porras J.S. (2013). Corangioma placentario: Enfoque clínico-patológico de un caso descrito en Colombia [Placental chorangioma: Clinic-pathological approach of a case in Colombia]. Ginecol. Obstet. Mex..

[171] Huang H.Q., Gong F.M., Yin R.T., Lin X.J. (2021). Choriocarcinoma misdiagnosed as cerebral hemangioma: A case report. World J. Clin. Cases.

[172] Webb S.D., Bonasoni M.P., Palicelli A., Comitini G., Heller D.S. (2022). Mixed chorangioma and leiomyoma of the placenta, with a brief review of nontrophoblastic placental lesions. Pediatr. Dev. Pathol..

[173] McMahon L.M., Joyce C.M., Cuthill L., Mitchell H., Jabbar I., Sweep F., on behalf of the hCG Working Party of the EOTTD (2024). Measurement of Human Chorionic Gonadotrophin in Women with Gestational Trophoblastic Disease. Gynecol. Obstet. Investig..

[174] Mandava A., Koppula V., Kandati M., Reddy A.K., Rajappa S.J., Rao T.S. (2024). Multimodality Imaging in the Diagnosis and Staging of Gestational Choriocarcinoma. Indian J. Radiol. Imaging.

[175] Amin M.B., Edge S., Greene F. (2017). AJCC Cancer Staging Manual.

[176] Jacobson R.J., Morley J.E., Shires R., Boles D., Saffer D. (1976). Choriocarcinoma presenting as Jacksonian epilepsy. S. Afr. Med. J..

[177] Toscano-Zukor A.M., Wang X. (2007). Overt hyperthyroidism secondary to metastatic gestational trophoblastic neoplasm. Endocrinologist.

[178] Fierro V., Freeman J.S. (1988). Choriocarcinoma-induced thyrotoxicosis: Report of a case and review of the literature. J. Am. Osteopath. Assoc..

[179] Godden J.D., Garnett E.S., Sommerville I.F., Bagshawe K.D. (1967). The effect of choriocarcinoma on serum thyroid hormone-binding capacity. J. Endocrinol..

[180] Rahmadhona D., Tambunan B.A. (2020). Gestational Trophoblastic Neoplasia with Hyperthyroidism. Indones. J. Clin. Pathol. Med. Lab..

[181] Sindiani A., Obeidat B., Alshdaifat E. (2020). Successful Management of the First Case of a Metastasized Complete Mole in Form of Twin Pregnancy in Jordan. Am. J. Case Rep..

[182] Alpay V., Kaymak D., Erenel H., Cepni I., Madazli R. (2021). Complete Hydatidiform Mole and Co-Existing Live Fetus after Intracytoplasmic Sperm Injection: A Case Report and Literature Review. Fetal Pediatr. Pathol..

[183] Odedra D., MacEachern K., Elit L., Mohamed S., McCready E., DeFrance B., Wang Y. (2019). Twin pregnancy with metastatic complete molar pregnancy and coexisting live fetus. Radiol. Case Rep..

[184] Eagan D., Jeter N. (2016). Complete molar pregnancy with transformation to choriocarcinoma of the liver: A case report. Case Rep. Women’s Health.

[185] Sasaki Y., Ogawa K., Takahashi J., Okai T. (2012). Complete hydatidiform mole coexisting with a normal fetus delivered at 33 weeks of gestation and involving maternal lung metastasis: A case report. J. Reprod. Med..

[186] Ji Y.I., Jung M.H. (2010). Gastrointestinal bleeding caused by ileal metastasis of a tubal complete mole: A case report. J. Women’s Health.

[187] Agrawal A., Agrawal C.S., Kumar A., Kumar M., Yadav R. (2007). Spontaneous acute subdural haemorrhage, cerebral and pulmonary metastases in a complete mole. Singap. Med. J..

